# Transition From Diencephalic Syndrome to Hypothalamic Obesity in Children With Suprasellar Low Grade Glioma: A Case Series

**DOI:** 10.3389/fendo.2022.846124

**Published:** 2022-04-06

**Authors:** Ichelle M. A. A. van Roessel, Antoinette Y. N. Schouten-van Meeteren, Lisethe Meijer, Eelco W. Hoving, Boudewijn Bakker, Hanneke M. van Santen

**Affiliations:** ^1^Department of Pediatric Endocrinology, Wilhelmina Children’s Hospital, University Medical Center Utrecht, Utrecht, Netherlands; ^2^Department of Pediatric Oncology, Princess Máxima Center for Pediatric Oncology, Utrecht, Netherlands; ^3^Department of Neurosurgery, Princess Máxima Center for Pediatric Oncology, Utrecht, Netherlands

**Keywords:** diencephalic syndrome, hypothalamic obesity, suprasellar tumor, Low Grade Glioma (LGG), hypothalamic pituitary

## Abstract

**Background:**

Children with suprasellar low grade glioma (LGG) frequently develop problems to maintain their body weight within the normal range, due to hypothalamic dysfunction. Hypothalamic damage may result in the diencephalic syndrome (DS), characterized by underweight or failure to thrive, but also in hypothalamic obesity (HO). Children with LGG presenting with DS at young age often develop HO later in life. The underlying pathophysiology for this change in body mass index (BMI) is not understood. Previous hypotheses have focused on the tumor or its treatment as the underlying cause. To better understand its etiology, we aimed to relate changes in BMI over time in children with suprasellar LGG presenting with DS to age, tumor progression, treatment, and endocrine function. We hypothesize that the development of HO in children with LGG presenting with DS is related to maturation status of the hypothalamus at time of injury and thus age.

**Methods:**

In this retrospective case series, all cases diagnosed in the Netherlands with suprasellar located LGG, currently treated or followed, with a history of DS developing into HO were included.

**Results:**

In total, 10 children were included. Median age at LGG diagnosis was 1.5 years (range 0.4–5.5), median BMI SDS was −2.64. The children developed overweight at a median age of 4.5 years (2.2–9.8). The median total difference in BMI SDS between underweight and obesity was +5.75 SDS (4.5–8.7). No association could be found between transition of DS to HO and onset of a pituitary disorder (present in 70.0%), surgery, chemotherapy, or tumor behavior. Two had developed central precocious puberty (CPP), both while having underweight or normal weight.

**Conclusion:**

The shift from DS to HO in children with hypothalamic LGG may be associated with age and not to tumor behavior, treatment characteristics or pituitary function. The development of CPP in these children seems not to be related to obesity. Our findings may indicate that the clinical picture of hypothalamic dysfunction reflects the maturation state of the hypothalamus at time of lesioning. Future prospective studies are needed to better understand underlying causative mechanisms of the morbid changes in body weight.

## Background

The hypothalamus, located in the suprasellar region of the brain, is a key regulator of energy homeostasis, feeding behavior and body composition (1, 2). Children with a tumor in the suprasellar region frequently develop problems in maintaining body weight within the normal range.

The most common described suprasellar tumor to affect body mass index (BMI) is the craniopharyngioma, leading to hypothalamic obesity (HO) in 75% of patients, of which a great percentage develop morbid obesity (1, 3). Childhood obesity correlates with morbidity and mortality at a later age (4). Aside from craniopharyngioma, suprasellar germinoma or low grade glioma (LGG) may also give rise to hypothalamic dysfunction, resulting in HO, with or without anterior pituitary dysfunction including some with diabetes insipidus (DI). Of children with LGG located along the optic pathway or in the suprasellar region, 50% ultimately develop obesity (5). At the other end of the spectrum are young children with LGG that present with underweight, which is also known as the diencephalic syndrome (DS) (6–9).

DS is characterized by loss of subcutaneous fat and failure to thrive with intact linear growth. Most children with DS are diagnosed within the first two years of life, at a mean age of six months, however outliers till ages up to sixteen years have been observed, mostly in children with optic pathway glioma (OPG) partially associated with Neurofibromatosis Type 1 (NF1) (6, 10–12).

The underlying pathophysiology for DS is not fully understood. Current hypotheses for weight loss focus on growth hormone (GH) overproduction with partial GH resistance, increased lipolysis by B-lipotropin and an elevated energy expenditure (6, 13, 14). Children presenting with DS can evolve into children with HO. Many questions still exist about the underlying causative mechanisms for this phenomenon and the sequence of development. Some propose that weight gain in children with DS is the result of increasing hypothalamic damage caused by specific tumor-treatment (15). While others indicate that the decrease in tumor size corresponds with weight gain, suggesting that the tumor itself is responsible for the weight loss (13). Reasonably, weight gain can also be the result of continued oral nutritional supplements or high caloric feeding as treatment for emaciation in DS.

The hypothalamus matures during childhood and hypothalamic dysfunction may result in different outcomes, depending on age (10, 13). Studies, mostly performed in rodents, have revealed insights in the development of the hypothalamus, and exposed the nuclei considered most important for satiety, feeding and metabolism, tuberal lateral nucleus, ventromedial hypothalamus, paraventricular nucleus, and arcuate nucleus (16, 17). It has been speculated that the sensitivity for brain injury due to tumor growth is related to immaturity of the hypothalamus, reflected by the fact that DS mostly develops within the first year of life (12).

Still, it is currently not known why some children with LGG or in rare cases, craniopharyngioma, develop DS while others develop HO. It is not sufficiently clear to what extent this is related to age, tumor-specific characteristics, endocrine dysfunction or hypothalamic immaturity. Furthermore, long-term longitudinal data on BMI development and the transition of DS to HO in children with LGG are scarce (10).

Brauner et al. correlated BMI SDS in children with DS to leptin, ghrelin, and hypothalamic–pituitary dysfunction. Out of the 11 children presenting with underweight in their review, eight developed obesity. It was observed that weight gain occurred in all patients with a decrease in tumor size after surgery, radiotherapy, or chemotherapy, while patients lost weight after recurrence of the tumor. In this cohort, central precocious puberty (CPP) was present in all but one case. It was proposed that CPP in these children was the result of early maturation of the gonadal system due to high estrogen and leptin levels caused by a high body fat mass (13). Rather than the result of obesity, however, CPP can also be the result of increased intracranial pressure affecting the inhibition of gonadotropin releasing hormone (GnRH) in the hypothalamus (18).

We hypothesize that: 1) DS in children with LGG is caused by hypothalamic damage due to the location of the tumor. The transition of DS to HO is related to age and correlates with (age-dependent) maturation of the hypothalamus, 2) CPP in these children is the direct consequence of activation of the GnRH pulse generator by increased pressure due to tumor location or hydrocephalus and not a consequence of obesity, 3) Pituitary deficiencies are prevalent in children who develop HO.

To study these hypotheses and to gain more insight into the pathophysiology of hypothalamic dysfunction in children with LGG, we aimed to describe the course of BMI over time in a case series of children presenting with the DS and who developed HO during follow-up. In addition, we aimed to describe the relation between changes in BMI and the following factors: age, tumor progression, treatment, and pituitary function.

## Methods

### Study Design and Population

We reviewed the medical records of all children with LGG who visited the Princess Máxima Center for pediatric oncology between June 2018 and May 2021 (*n = 429*). Children with a suprasellar LGG and a history of DS who subsequently developed HO were included and described.

### Data Collection

Data were collected by retrospective chart review. Follow-up data was collected until May 2021.

## Operational Definitions

### Diencephalic Syndrome (DS) and Hypothalamic Overweight or Obesity (HO)

DS was defined as having a BMI SDS <−1.6 SD or weight/height ratio (corrected for age and gender) <−1.6 SD or if the medical history mentioned failure to thrive (described as such by attending physician). Children with a duration of having underweight less than 2 months were excluded from analysis.

(Hypothalamic) overweight or obesity (HO) was defined as BMI SDS >+1.6 SD or weight/height ratio (corrected for age and gender) >+1.6 SD.

### Pituitary Disorders

Diagnostic criteria for the presence of an endocrine disorder were in accordance with our previous study (19).

Pituitary disorder was defined as presence of any anterior pituitary hormone deficiency, CPP or DI. Anterior pituitary hormone deficiency included presence of growth hormone deficiency (GHD), thyroid-stimulating hormone deficiency (TSHD), luteinizing hormone and follicle-stimulating hormone deficiency (LH/FSHD), or adrenocorticotropic hormone deficiency (ACTHD). CPP was defined as Tanner B2 in girls below the age of eight years or testes volume ≥4 ml in boys under the age of nine, in combination with detectable LH or FSH concentrations, a peak LH >5 IU/L in response to GnRH or use of GnRH analogue.

IGF-1 overproduction was defined as IGF-1 SDS (by age and gender) ≥2 SD.

### Resting Energy Expenditure (REE)

If available, data was collected upon measurements for determination of REE. For this purpose, indirect calorimetry had been used in fasting individuals. Calculated REE as percentage of predicted REE between 90 and 110% for sex, age and weight, according to Schofield equation, was considered normal (20).

### Time Periods

Changes in BMI, pituitary function, tumor- and treatment characteristics were assessed for the following time periods: the period underweight to normal weight (UW–NW), the period normal weight to overweight (NW–OW), and the period overweight to obesity (OW–OB).

**Figure 1:**
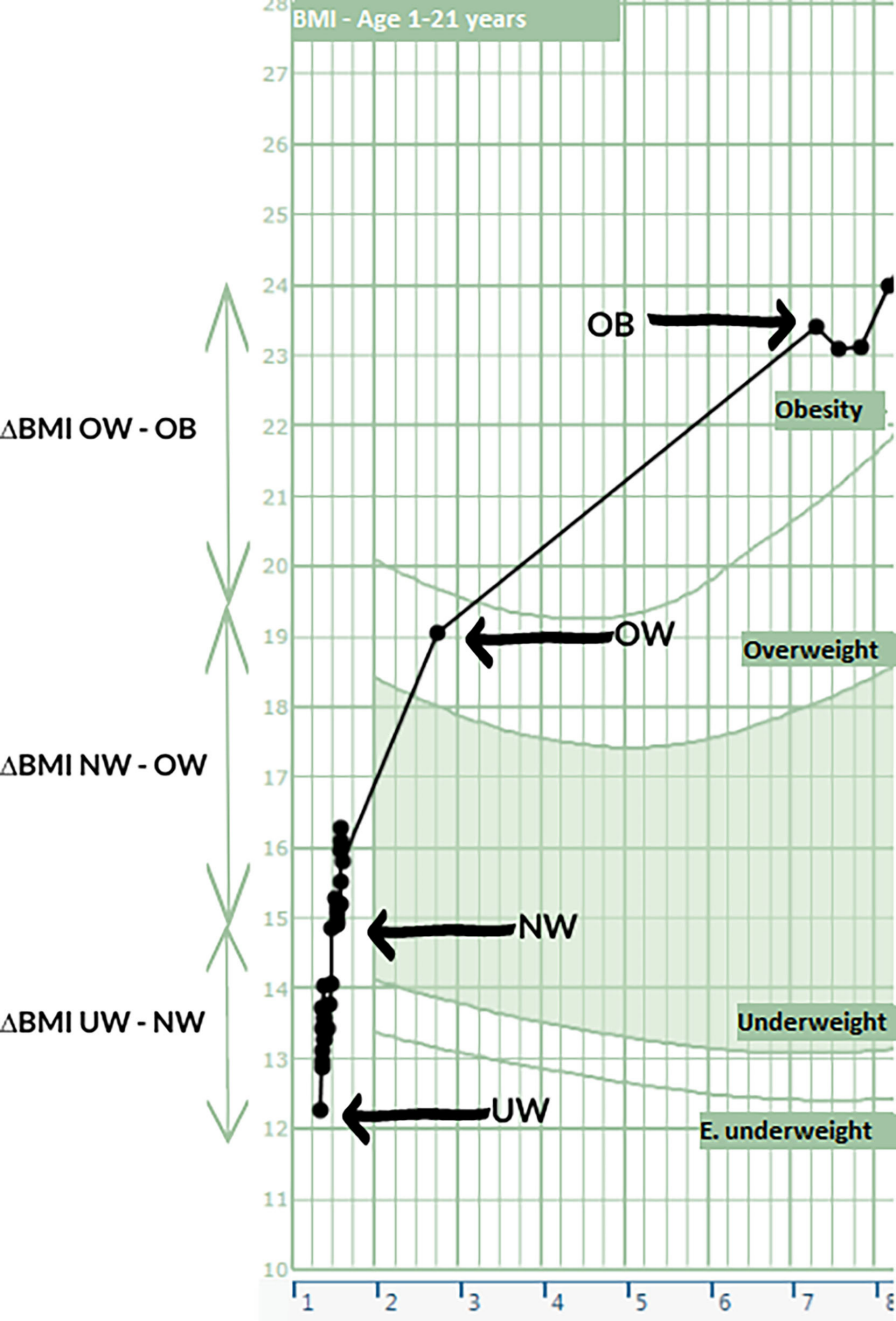
Case of DS evolving into HO to support **Table 2**. On the X axis the age is shown and on the Y axis the BMI. Various time points as displayed in **Table 2** are shown here.

UW–NW was defined as the time between the first measurement of underweight at the time of tumor diagnosis to the first measurement of BMI within normal range. NW–OW was defined as the time between the first measurement of NW to the first measurement of OW. OW–OB was defined as the time between the first measurement of OW to the first measurement of OB (see **Figure 1**).

### Other Definitions

Tumor histology was defined as pilocytic astrocytoma or pilomyxoid astrocytoma.

Hydrocephalus was defined as diagnosis of hydrocephalus on the MRI report or history of hydrocephalus corrective surgery (drain, shunting, ventriculostomy).

Follow-up time was defined as the time between date of diagnosis and last hospital appointment.

Tumor neurosurgery was considered as partial resection, debulking, or total resection. Biopsy as procedure and correction of hydrocephalus by ventriculoperitoneal shunt or ventriculostomy were assessed as separate procedures but not included as surgical treatment.

Tumor growth was defined as the increase of tumor size as measured and reported by radiologist on MRI findings, or if correspondence in the medical chart mentioned progression of tumor.

Chemotherapy was administered according to the International Society of Pediatric Oncology-Low Grade Glioma trial protocols (SIOP LGG) (carboplatin vincristine for 1.5 years) or local treatment guidelines in absence of an open study.

### Statistical Analyses

Statistical analyses were performed using the SPSS statistical software version 26.0 (IBM, United States).

For all patients, demographic characteristics (age, gender, follow-up time), tumor-related characteristics (histology, location, state of disease, relapse, metastases, hydrocephalus), treatment-related characteristics (surgery, chemotherapy, radiotherapy) and endocrine data were collected and described.

Longitudinal data on BMI and IGF-1 were analyzed using the R version 1.3.1093 (R core team, Austria) with the following packages: ggplot2, lme4, dyplyr, grid, gridExtra, car. Local regression curves (LOESS) with 95% confidence intervals were plotted. This is a nonparametric method for data exploration without assumptions. To test whether using a nonparametric method was correct, we have also provided the individual curves. QQ-plots were used to test for normality. Kendall rank correlation test was used to estimate measure of association between BMI SDS and IGF-1 SDS.

## Results

### Study Population

In total, 10 children were included. All children had been diagnosed with a suprasellar located LGG, of which seven were pilocytic astrocytoma (70.0%), and in three children with NF1, data on histology was not available. Median age at diagnosis was 1.5 years (range 0.4–5.5). Median follow-up time was 8.1 years (range 3.1–12.4). All but one had been treated with chemotherapy. Six (60.0%) of the 10 patients had undergone surgery, whereas none of the children had been given radiotherapy. Patient characteristics are summarized in **Table 1**.

**Table 1:** Descriptive characteristics of the 10 patients.

Characteristic		N
**Female gender**	40.0%	4/10
**Age at diagnosis, years, median (IQR)**	1.46	0.58–3.31
**Age at follow-up, years, median (IQR)**	9.74	5.81–11.25
**Follow-up time, years, median (IQR)**	8.10	4.63–9.42
**Histology**		
– **Pilocytic astrocytoma**	70.0%	7/10
**–** **No information available**	30.0%	3/10
**NF 1**	30.0%	3/10
**Metastases at diagnosis**	20.0%	2/10
**Hydrocephalus**	40.0%	4/10
**Endocrine disorder at diagnosis**	0.0%	0/10
**Relapse**	80.0%	8/10
**Surgery**	60.0%	6/10
**Number of tumor surgeries, median (range)**	1	0–5
**Chemotherapy**	90.0%	9/10
**Rounds of chemotherapy, median (range)**	2	0–7
**Radiotherapy**	0.0%	0/10
**State of disease at follow-up**		
– **Stable residual disease**	70.0%	7/10
– **Progressive disease but clinically well**	30.0%	3/10

### BMI Over Time

For seven children data on birth weight were available. From these children, six were normal weight at birth, and one was underweight at birth, with weight below the 3th percentile. This child was born term. Median BMI SDS at underweight was −2.64 SDS. After a median period of 11 months (range 2.2–18.4 months), BMI was within normal range (UW-NW). Transition to overweight (NW-OW) occurred at a median age of 4.5 years (median BMI SDS +2.00), followed by development of obesity at a median age of 5.1 years (median BMI SDS +2.77). The median of the highest BMI reached was 24.0 kg/m^2^ (range 20.36–32.16) (+3.32 SDS). The median maximal difference in BMI SDS between underweight and obesity (Max Δ BMI SDS) was +5.75 SDS (range 4.49–8.72). At follow-up, only one child was normal weight (BMI SDS +1.54), whereas 9 children (90.0%) were overweight or obese (**Table 2**).

**Table 2:** Change in BMI over time.

Nr	Age at Dx, yrs	Age UW	BMI SDS	ΔBMI UW–NW	Age NW	ΔBMI NW–OW	Age OW	BMI SDS	ΔBMI OW–OB	Age OB	BMI SDS	Max Δ BMI SDS*
A	1.3	**1.3**	−4.76	3.01	**1.5**	3.78	**2.7**	2.20	4.35	**7.3**	3.76	8.52
B	0.5	**0.5**	−2.97	2.05	**2.0**	3.49	**4.6**	2.04	1.41	**5.1**	2.69	6.29
C	0.4	**0.4**	−2.46	1.82	**1.5**	3.83	**2.3**	1.95	0.55	**2.5**	2.46	5.75
D	1.6	**1.2**	−2.81	2.03	**1.6**	4.06	**2.2**	2.51	–	**2.2**	2.51	6.77
E	2.1	**2.5**	−3.59	1.67	**3.4**	3.55	**4.9**	1.91	–	**–**	–	–
F	1.0	**1.0**	−1.74	0.85	**2.2**	1.78	**4.9**	1.86	1.69	**5.3**	2.82	5.04
G	2.9	**3.0**	−1.73	0.22	**3.3**	3.98	**3.9**	2.09	0.87	**4.5**	2.68	5.49
H	0.6	**0.9**	−4.07	2.92	**1.9**	4.21	**4.4**	3.08	0.59	**4.5**	3.37	8.72
I	5.5	**3.8**	−1.72	–	**–**	–	**5.5**	1.68	10.24	**12.4**	2.77	4.49
J	4.5	**7.1**	−1.88	0.48	**7.5**	7.01	**9.8**	1.79	9.84	**16.0**	2.85	5.01
Median	1.5	**1.3**	−2.64	1.82	**2.0**	3.83	**4.5**	2.00	0.65	**5.1**	5.77	5.75

Dx, diagnosis.

NW, normal weight (first measurement of BMI within normal range).

OB, obesity (first measurement of obesity).

OW, overweight (first measurement of overweight).

UW, underweight (first measurement of underweight at time of tumor diagnosis).

E. underweight, extreme underweight.

*BMI SDS difference between maximum obesity and underweight.

Vertical lines represent a period of 3 months and horizontal lines represent 1 BMI kg/m^2^.

Bold values distinguish the various time periods.

**Figure 2:**
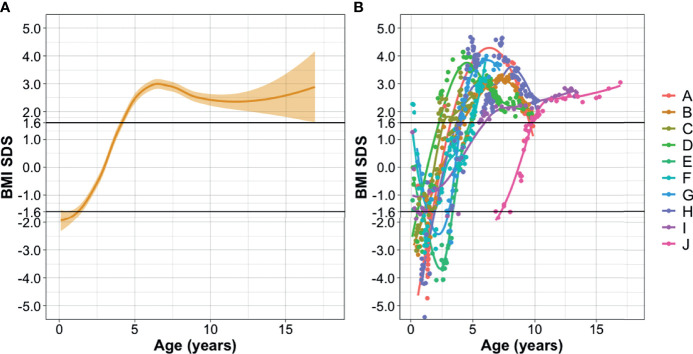
Longitudinal BMI SDS over time. **(A)** Logistic regression (LOESS) plot of all patients. x-axis: age in years at moment of BMI follow-up, y-axis: BMI SDS corrected for age and gender. Orange beam shows 95% confidence interval. **(B)** Individualized BMI SDS data for each patient. This figure shows that the regression curve represents most of the patients, with only two outliers observed. Diencephalic syndrome defined as BMI SDS <−1.6 SD. Overweight defined as BMI SDS >+1.6 SD. Legend shows patients in correspondence with **Table 2**.

Longitudinal BMI SDS over time is shown in **Figure 2**.

### Pituitary Disorders

**Figure 3:**
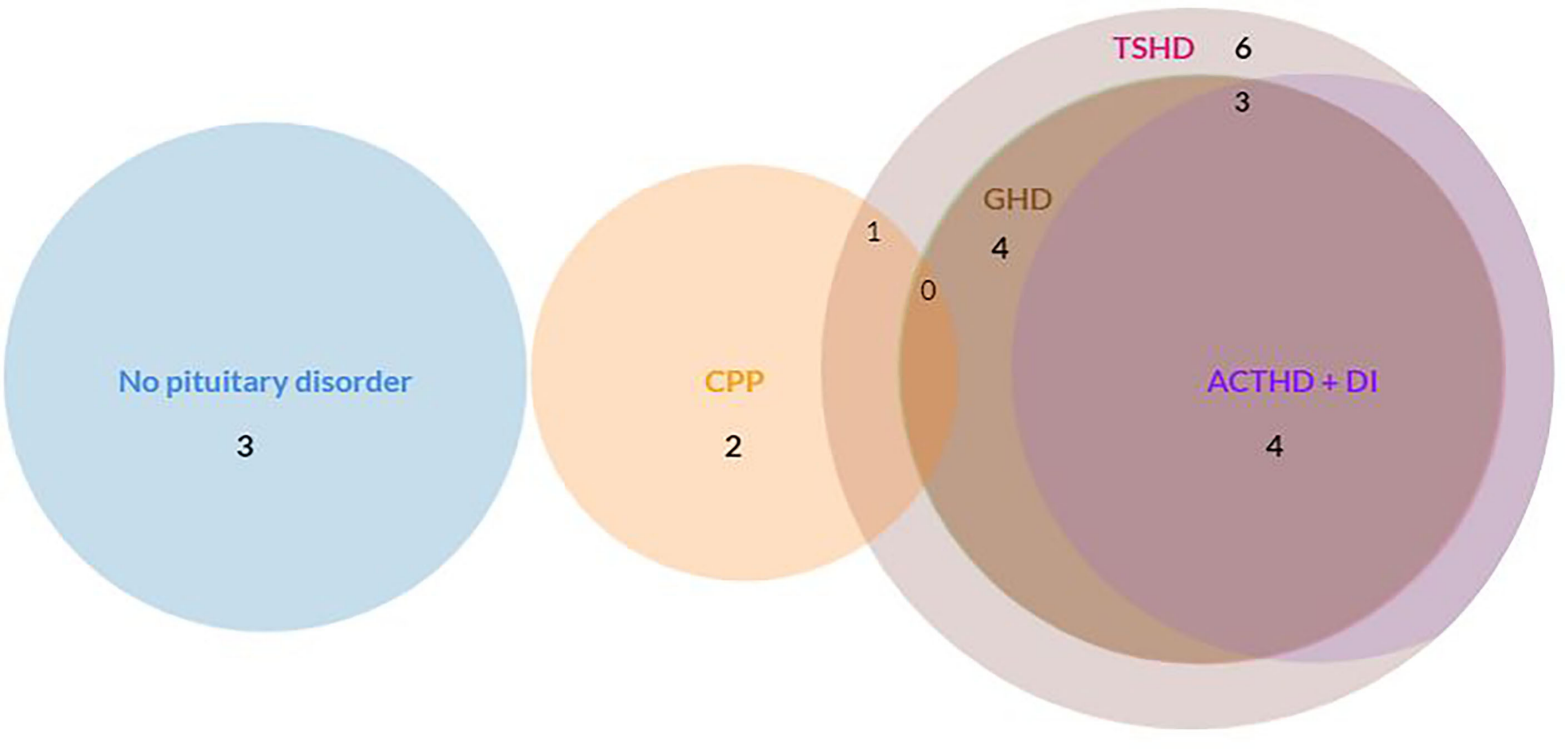
Prevalence of pituitary disorders (anterior pituitary deficiency, CPP, DI). ACTHD, adrenocorticotropic hormone deficiency; CPP, central precocious puberty; DI, diabetes insipidus; GHD, growth hormone deficiency; TSHD, thyroid-stimulating hormone deficiency. The circles of ACTHD and DI are completely overlapping: every child with ACTHD also had DI. None of the children had LH/FSHD. N = 3 had no pituitary disorder. N = 1 had CPP. N = 1 had CPP and TSHD. N = 1 had GHD and TSHD. N = 1 had TSHD, ACTHD and DI. N = 3 children had GHD, TSHD, ACTHD, and DI.

None of the children had been diagnosed with a pituitary disorder at tumor diagnosis. In total, seven children developed a pituitary disorder (70.0%) during follow-up, of whom six developed an anterior pituitary deficiency (60.0%). Prevalence of all pituitary disorders is shown in **Figure 3**.

### Treatment and Pituitary Hormone Deficiencies in Relation to Changes in BMI

Timeline of events for each transition period is shown in **Table 3**.

**Table 3:** Timeline of events, treatment and pituitary deficiencies in relation to BMI.

Nr	Age at Dx	Age UW	Sx	CT	TG	Pit def	PP	DI	Age NW	Sx	CT	TG	Pitdef	PP	DI	Age OW	Sx	CT	TG	Pitdef	PP	DI	Age OB
A	1.3	1.3							1.5				**==**			2.7				**==**			7.3
B	0.5	0.5							2.0					**==**		4.6				**==**	**==**		5.1
C	0.4	0.4	B						1.5							2.3		T		**==**			2.5
D	1.6	1.2			?				1.6							2.2				*			2.2
E	2.1	2.5	B						3.4							4.9	–	–	–	–	–		–
F	1.0	1.0	B						2.2							4.9				**==**			5.3
G	2.9	3.0			?				3.3			?				3.9							4.5
H	0.6	0.9							1.9				**==**			4.4				**==**			4.5
I	5.5	3.8							–							5.5			?				12.4
J	4.5	7.0			?				7.5			?				9.8			?				16.0
**Time during follow-up**																							
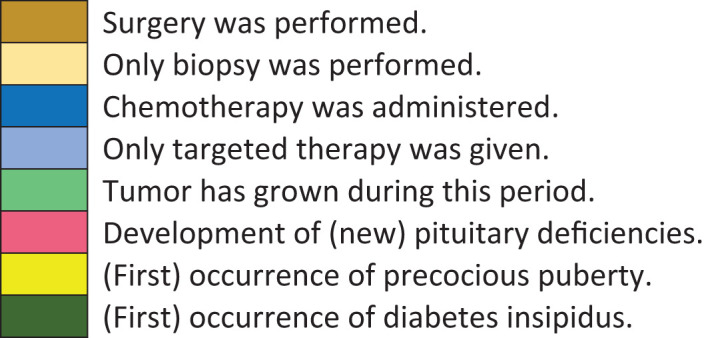																							

B, biopsy only; Dx, diagnosis; CT, chemotherapy; OB, obesity; OW, overweight; Pit. def, pituitary deficiency; PP, precocious puberty; Sx, surgery; T, targeted therapy only; TG, tumor growth; UW, underweight.

Color-filled boxes correspond with occurrence of events during that time period for that child.

Tumor treatment between underweight and normal weight, normal weight and overweight, and overweight and obesity are shown.

None of the children had been given radiotherapy.

==already had pituitary insufficiency, without further development of new insufficiencies.

* developed pituitary deficiency while having obesity.

? no data available.

Most of the children underwent tumor neurosurgery while having underweight or normal weight. Chemotherapy was given during all time periods. One child was not treated with chemotherapy.

Tumor growth occurred in the periods from transition UW-NW and in NW-OW. With recurrence of tumor, no weight loss was observed. In four children tumor size remained stable during follow-up.

Children developed pituitary hormone deficiencies in the periods of transition UW-NW and NW-OW. None of the children had experienced weight gain within one month after start of hydrocortisone.

In total, four children had hydrocephalus, two of whom presented with hydrocephalus at tumor diagnosis. One developed hydrocephalus while having normal weight, and the other one while being overweight. Three out of four children had hydrocephalus surgery.

### CPP and Obesity

CPP was present during follow-up in two children (20.0%) at ages of 0.8 and 4.3. Both were female. In one, CPP developed during UW-NW while the other developed CPP while having a normal weight (**Table 3**). One had previous hydrocephalus.

### Resting Energy Expenditure

In 8 out of 10 children, REE measurements were performed after the development of overweight or obesity. In seven children (87.5%) measured REE (mREE) values were below 90% of predicted (three children <60%, two children < 80%, two children < 90%). mREE values were between 90 and 110% of predicted values in only one child.

In none of the children, REE measurements at time of underweight or during normal weight were available.

### Other Impairments

In seven children visual acuity deficits were observed, with four of the children experiencing severe vision loss (40.0%) and three of the children being completely blind (30.0%). In four of the 10 children, visual field loss (hemianopsia) was detected. Two of the 10 children did not experience any visual problems (no visual acuity- or visual field defects).

In two children, the severe comorbidity led to impaired mobility with a requirement of wheelchair use.

In 60.0% (6/10) of the children, sleep disturbances were documented and in three children (30.0%) a temperature regulation disorder was present with frequent periods of hypothermia.

### IGF-1 Overproduction

**Figure 4:**
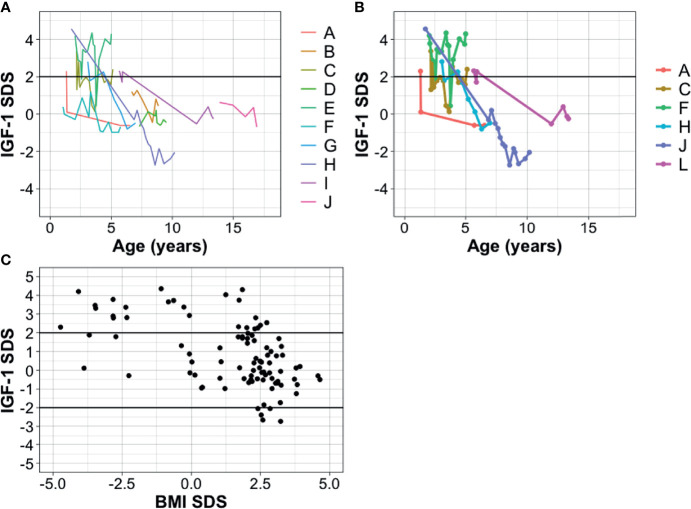
Longitudinal IGF-1 SDS over time in children with suprasellar LGG presenting with DS. Legend shows patients in correspondence with **Table 2** and **Figure 2**. **(A)** IGF-1 SDS values over time in all patients. x-axis: age in years at time of measurement, y-axis: IGF-1 SDS corrected for age and gender. **(B)** IGF-1 SDS values over time for patients with GH hypersecretion only (for 1 patient with GH hypersecretion, data on IGF-1 values over time were missing). **(C)** BMI SDS (x-axis) according to IGF-1 SDS (y-axis) in all patients. Correlation coefficient for BMI-SDS and IGF-1 SDS: −0.37 (p <0.001). Only IGF-1 values without growth hormone treatment or use of somatostatin analogues are shown.

In seven out of 10 children (70.0%) IGF-1 levels were elevated (IGF-1 ≥2 SDS, by age and gender) at time of diagnosis or during follow-up. In two of these seven children, a spontaneous normalization of IGF-1 was observed (IGF-1 SDS 0.4 and −0.5, respectively) whereas in two children IGF-1 levels were elevated at follow-up (IGF-1 SDS 2.4 and 4.3, respectively, after 5.1 years and 5.0 years of follow-up time respectively). In one child treatment with somatostatin analogues was initiated, which resulted in normalization of IGF-1 levels. During follow-up, in two children a transition of IGF-1 overproduction to growth hormone deficiency was observed. One of them was treated for GHD and the other child had an IGF-1 value of −2.1 at follow-up. **Figure 4** shows course of IGF-1 values over time in all patients and in patients with GH hypersecretion.

### IGF-1 and BMI SDS

Children with underweight were found to have higher levels of IGF-1 (median 2.8 SDS in underweight children versus 0.20 SDS in normal-overweight children). BMI SDS was found to be inversely related to the level of IGF-1, with a correlation coefficient of −0.38 (p <0.001).

## Discussion

In this small case-series, retrospectively evaluated, of children with suprasellar LGG presenting with DS, we aimed to describe the clinical course of DS to HO and related this to age, tumor- and treatment-specific characteristics, and pituitary function. Susceptibility of hypothalamic neurons may be related to (im)maturation status and therefore damage to the hypothalamus may result in different clinical outcomes depending on age. Our case series seems to demonstrate that the crucial transition from DS to HO occurs at a median age of 4.5 years. As this occurred at approximately the same age in most children, this may indeed support the hypothesis that this transition is related to maturation state of the hypothalamus.

We chose to include children with suprasellar located LGG in this study. These children are at risk for hypothalamic damage due to tumor location, as has also been described in children with suprasellar extension of craniopharyngioma (21). We found no unanimous denominator in the events during the distinct weight transition periods.

Previous authors have suggested that surgery or chemotherapy were the causative of weight gain in children with DS (15, 22) but we could not confirm this association in our case series. Of the children developing HO after DS 60% had undergone surgery and one child who developed HO had not received surgery or chemotherapy. All but one of the children in this cohort were given chemotherapy, however this was given during all transition periods, and could not be related with weight gain.

Also, no relationship between tumor progression and weight gain was observed in our case-series. In contrast to the findings of a previous study, weight loss in our cohort was not observed after tumor recurrence, suggesting that the tumor itself is not responsible for weight loss and weight gain is not the direct consequence of decrease in tumor size which has been suggested by others (13).

The change in BMI SDS score in this cohort with hypothalamic damage points towards an association of BMI to age and maturation status of the hypothalamus at time of lesioning. The role of the hypothalamus in feeding behavior and energy balance was revealed in the 1930s, when experimental lesions into the ventromedial nucleus were shown to result in hyperphagia, whereas lesions in the lateral nucleus resulted in hypophagia (23, 24). Swaab et al. showed that aging of cells in the hypothalamus follows a different pattern in various nuclei. Also, age was shown to impact the neuronal metabolic activity, wherein the metabolic activity of these neurons (assessed as number of Golgi apparatus per cell) declined significantly with age (25). In rats, it was shown that the mRNA expression levels of orexigenic peptides rise with age from the neonatal period to pre-puberty, whereas anorexigenic peptides decrease with age (26).

Secondly, we hypothesized that rather than the result of obesity, CPP in these children is the direct result of activation of the gonadal axis due to damage to the HP region by the tumor or increased intracranial pressure (hydrocephalus). Others have suggested that, since CPP is prevalent in children with hypothalamic obesity, CPP is the result of increased body fat mass and subsequent estrogen and leptin production, causing early maturation in the child (13). In rat studies, it was shown that leptin levels remain constant in the prepubertal and postpubertal stages and leptin gene expression in the hypothalamus did not show any developmental change. Therefore, leptin seems to act as permissive for onset of puberty but may not be the most important metabolic trigger (27). In our case-series, the children with CPP developed CPP before they became overweight or obese, which contradicts it being a result of obesity, estrogen or leptin. The pituitary gland produces LH and FSH after stimulation of GnRH from the hypothalamus (28). In prepubertal children, LH and FSH are low due to tonic inhibition of GnRH, possibly by GABA neurons. This pulse generator can be “activated” by brain damage (caused by the tumor itself or treatment) or increased intracranial pressure, such as in children with spina bifida or after meningitis (29). In our case-series also one of the children had hydrocephalus. Given the low number of children with CPP, these results should be confirmed in future studies.

Weight gain in children with hypothalamic damage may be accompanied by anterior pituitary deficiencies, which was also confirmed in a recent study (30). Hypothalamic tumor involvement and DS were found to be independent risk factors for anterior pituitary dysfunction previously (5). As was shown by Müller et al., patients with panhypopituitarism do not experience any problems with maintaining weight within normal range if no hypothalamic involvement was observed (21). In our cohort, nearly half of the patients who developed obesity developed an anterior pituitary deficiency suggesting that obesity in these children was caused by hypothalamic dysfunction but not by pituitary deficiencies.

Previous studies reported elevated levels of GH and IGF-1 in patients with DS and considered dysregulation of GH causative for weight loss and emaciation, with mobilization of free fat mass (13, 31, 32). GH hypersecretion was thought to be induced by hypercortisolism and ghrelin production, comparable to the pathomechanism of anorexia nervosa (31). In our patients, GH measurements were not performed, but IGF-1 overproduction was present in 7/10 patients. Interestingly IGF-1 values were found to be inversely related to BMI SDS. This dysregulation of the GH-axis in DS should be explored further. Next to GH, also leptin, ghrelin and oxytocin are thought to be dysregulated in hypothalamic obesity (13, 16, 17, 33). Changes in the secretion of these hormones may result in the evolution of DS to HO.

The magnitude of the change of BMI in children included in this case-series was impressive. Also the timespan in which underweight changed to overweight was short, with BMI in the normal range after eleven months median. It may be questioned whether the active high caloric feeding which we gave such children is favorable or that we should aim for a slower normalization of BMI. We may even hypothesize, that underweight and feeding problems serve as a protective mechanism to prevent further increase of the level of IGF-1 and may influence subsequent tumor growth. If this were true, it may even be considered that underweight is not only pathological but also an evolutionary phenomenon to protect the child from tumor growth.

The fact that visual disturbances were prevalent in these children was not surprising, given the close location of the tumor in the optic pathway or optic chiasm. In two children, severe morbidity even resulted in the impaired mobility and subsequent the use of a wheelchair. The decrease of physical activity following these impairments of course contributes significantly to weight gain in these children.

To distinguish whether increase in BMI was the result of hypothalamic damage, resting energy expenditure measurements were performed in almost all children. Out of 8 children that were measured while having overweight or obesity, seven had a lower REE than predicted according to gender and age. Two of these eight children were bound to a wheelchair. It must be considered that REE is decreased due to diminished muscle mass. However, the fact that in both of these children many other signs of hypothalamic dysfunction were present (temperature dysregulation, panhypopituitarism, diabetes insipidus without thirst regulation, hyperphagia, and day–night disturbances), supports hypothalamic dysfunction be causative of the low REE.

In the child with normal REE measurement, hyperphagia was present among other symptoms of hypothalamic dysfunction such as impaired impulse control, temperature- and sleep dysregulation. These all point toward hypothalamic damage as underlying cause for weight gain, even though energy expenditure was not affected. Unfortunately, no measurements of REE were available at time of underweight or normal weight. In two previous studies REE in children with diencephalic syndrome was assessed. REE was 30–50% higher compared to healthy children and Kilday et al. even found REE values over 200% of predicted in children with DS (15, 34). These findings indicate that weight loss in DS may be the result of a hypermetabolic state. Treatment of tumor resulted in normalization of REE values (15).

### Strengths and Limitations

Our findings are limited by the retrospective nature of this study. Unfortunately, individual information on diet and physical activity was not available in retrospective chart review and could not be evaluated. Underweight at diagnosis could also reflect high intracranial pressure and subsequent vomiting resulting in weight loss, rather than the diencephalic syndrome. Given the small number of cases and the descriptive character of our case-series, it cannot be ruled out that treatment may have contributed to weight gain.

Most of the children included in this study were referred from another hospital, leading to incomplete data on endocrine parameters, determined in the past, including IGF-1 values. Despite our efforts to collect complete information from other centers, some data remained missing. Furthermore, BMI is not an ideal outcome as it cannot differentiate between fat and lean body mass. In literature, BMI is defined differently with various cut-off points for overweight and obesity. In this study, BMI SDS <−1.6 and >+1.6 were used to define underweight or overweight respectively since it corresponds with the 10th percentile (35). It should however be noted that these cut-off points for normal values are based on distribution curves in the population from 1980 where obesity was less prevalent (10.1% in 1980 *v* 15.1% in 2020) (36, 37).

Despite these limitations, the description of these cases has allowed us to give a detailed view of children with LGG with DS developing HO. It is the first case-series to relate granular longitudinal BMI data to age in children with DS and HO. An insight into the relation of DS and HO to endocrine and tumor- and treatment related characteristics is provided.

### Future Perspective

Our case series may form the basis for further exploration of the underlying pathophysiology of DS and HO in children with LGG. In the future, prospective studies upon leptin, ghrelin and oxytocin concentrations in children with DS and HO may help to better understand the underlying pathophysiology for DS and HO. Also, functional MRI studies may provide more detailed evaluation of specific hypothalamic nuclei in relation to the development of underweight or obesity. Additionally, future studies should prospectively include REE measurements and systematic hypothalamic dysfunction scoring to adequately access whether underweight and obesity are the result of hypothalamic dysfunction or other contributing factors such as decreased mobility or socio-economic factors (38). Long-term REE measurements may also be useful for counseling of diet and physical activity.

### Conclusion

DS in children with suprasellar LGG seems to transite to HO at a median age of 4.5 years, regardless of patient or treatment characteristics, which may be the consequence of hypothalamic dysfunction changing with age and maturation status in time. Contributing factors for increasing BMI such as visual impairment, decreased mobility, inadequate feeding pattern, disturbed day-night rhythm or socio-economic factors may be present. To better understand its etiology and develop ways to prevent or decrease obesity and its long term complications in children with suprasellar LGG, collaborative studies are needed including larger cohorts.

## Data Availability Statement

The raw data supporting the conclusions of this article will be made available upon request by the authors, without undue reservation.

## Ethics Statement

Written informed consent was obtained from the individual(s), and minor(s)’ legal guardian/next of kin, for the publication of any potentially identifiable images or data included in this article.

## Author Contributions

IR, AS, and HS contributed to conception and design of the study. IR organized the database, performed the statistical analysis and wrote the first draft of the manuscript. HS, AS, LM, EH, and BB wrote sections of the manuscript. All authors listed have made a substantial, direct, and intellectual contribution to the work and approved it for publication.

## Conflict of Interest

The authors declare that the research was conducted in the absence of any commercial or financial relationships that could be construed as a potential conflict of interest.

## Publisher’s Note

All claims expressed in this article are solely those of the authors and do not necessarily represent those of their affiliated organizations, or those of the publisher, the editors and the reviewers. Any product that may be evaluated in this article, or claim that may be made by its manufacturer, is not guaranteed or endorsed by the publisher.

## Abbreviations

ACTHD, adrenocorticotropic hormone deficiency; BMI, body mass index; CBTS, childhood brain tumor survivors; CPP, central precocious puberty; DI, diabetes insipidus; DS, diencephalic syndrome; FSH, follicle stimulating hormone; GH, growth hormone; GHD, growth hormone deficiency; GnRH, gonadotropin-releasing hormone; HO, hypothalamic obesity; HP, hypothalamic-pituitary; LGG, low grade glioma; LH, luteinizing hormone; LH/FSHD, luteinizing hormone and follicle-stimulating hormone deficiency; MREE, measured resting energy expenditure; NF1, Neurofibromatosis type 1; NW, normal weight; OB, obesity; OPG, optic pathway glioma; OW, overweight; REE, resting energy expenditure; TSHD, thyroid-stimulating hormone deficiency; UW, underweight.
